# Plant and fungal products that extend lifespan in *Caenorhabditis elegans*

**DOI:** 10.15698/mic2020.10.731

**Published:** 2020-07-09

**Authors:** Jan Martel, Cheng-Yeu Wu, Hsin-Hsin Peng, Yun-Fei Ko, Hung-Chi Yang, John D. Young, David M. Ojcius

**Affiliations:** 1 Center for Molecular and Clinical Immunology, Chang Gung University, Taoyuan, Taiwan.; 2 Chang Gung Immunology Consortium, Chang Gung Memorial Hospital at Linkou, Taoyuan, Taiwan.; 3 Research Center of Bacterial Pathogenesis, Chang Gung University, Taoyuan, Taiwan.; 4 Laboratory Animal Center, Chang Gung Memorial Hospital at Linkou, Taoyuan, Taiwan.; 5 Chang Gung Biotechnology Corporation, Taipei, Taiwan.; 6 Biochemical Engineering Research Center, Ming Chi University of Technology, New Taipei City, Taiwan.; 7 Department of Medical Laboratory Science and Biotechnology, Yuanpei University of Medical Technology, Hsinchu, Taiwan.; 8 Department of Biomedical Sciences, University of the Pacific, Arthur Dugoni School of Dentistry, San Francisco, CA, USA.

**Keywords:** autophagy, caloric restriction mimetics, dietary supplements, hormesis, phytochemicals

## Abstract

The nematode *Caenorhabditis elegans* is a useful model to study aging due to its short lifespan, ease of manipulation, and available genetic tools. Several molecules and extracts derived from plants and fungi extend the lifespan of *C. elegans* by modulating aging-related pathways that are conserved in more complex organisms. Modulation of aging pathways leads to activation of autophagy, mitochondrial biogenesis and expression of antioxidant and detoxifying enzymes in a manner similar to caloric restriction. Low and moderate concentrations of plant and fungal molecules usually extend lifespan, while high concentrations are detrimental, consistent with a lifespan-modulating mechanism involving hormesis. We review here molecules and extracts derived from plants and fungi that extend the lifespan of *C. elegans*, and explore the possibility that these natural substances may produce health benefits in humans.

## INTERVENTIONS TO DELAY AGING

Aging can be modulated by genes and lifestyle. For instance, specific gene variants of insulin-like growth factor-1 (IGF-1) receptor and forkhead box O3A (FOXO3A) are associated with longer lifespan in centenarians [1]. In terms of lifestyle, one of the most studied interventions that delay aging is caloric restriction (CR), which can increase lifespan in organisms ranging from yeasts to primates [2]. Diet composition also influences the aging process, with low-protein diets [3, 4] and high phytochemical intake [5, 6] being associated with a longer lifespan. Notably, a recent analysis suggests that the heritability of human longevity is below 10% [7], indicating that lifestyle choices play a major role in influencing aging and longevity.

Since interventions such as CR and dieting are difficult to implement and maintain over a long period, interest has focused on identifying molecules that produce effects similar to CR (i.e., the CR mimetics). This endeavor is based on the observation that signaling pathways that are modulated by CR, including 5' adenosine-monophosphate-activated protein kinase (AMPK), mammalian target of rapamycin (mTOR) and sirtuin-1, can be targeted by small organic compounds [8]. Activation of these pathways induces autophagy, mitochondrial biogenesis and expression of antioxidant and detoxifying enzymes, which together can improve cellular function [2, 9, 10]. In a manner similar to CR, several organic compounds labeled as CR mimetics promote physiological functions and reduce the development of chronic diseases, thus improving both health and longevity [8].

**Figure 1:**
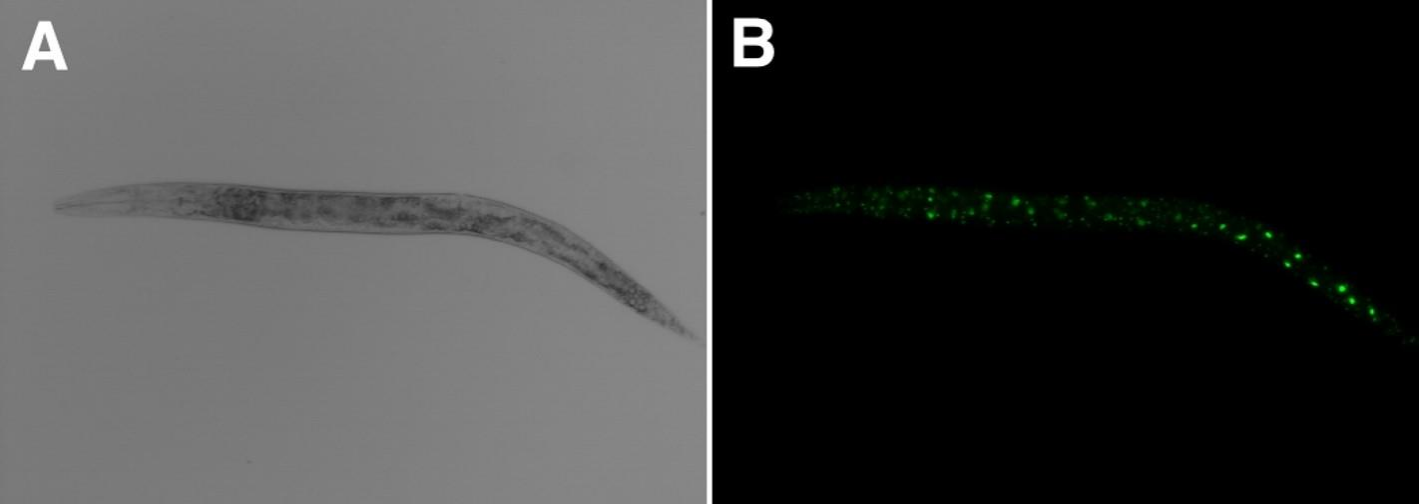
FIGURE 1: Images of *C. elegans* nematode used as a model to study aging and longevity. **(A)** Light microscopy and **(B)** fluorescence microscopy images of transgenic *C. elegans* strain CGUIS-1 expressing the nucleolar protein fibrillarin 1 (FIB-1) coupled to green fluorescent protein (GFP). FIB-1 is a marker of nucleolus size that negatively correlates with longevity across taxa [161], making the CGUIS-1 strain useful for screening natural products that may extend lifespan. In B, GFP auto-fluorescence is induced by ultraviolet light. The images are unpublished observations made by the authors.

The nematode *Caenorhabditis elegans* is a useful model organism for studying aging [11] (**Figure 1**). One of the main advantages of *C. elegans* is its short lifespan of about 20 to 25 days, allowing the rapid screening of substances that affect longevity. In addition, nematodes can be manipulated easily and single-gene deletion mutants are readily available, which facilitates the identification of signaling pathways involved in lifespan extension. Furthermore, many cellular pathways that control aging in *C. elegans* are conserved in more complex organisms, including fruit flies, mice and humans [12]. Modulation of the gut microbiota can also positively or negatively influence health and longevity in *C. elegans* [13, 14]. We review here the molecules and extracts derived from plants and fungi that are known to extend the lifespan of *C. elegans*, and discuss the possibility of using these substances in humans.

## PLANT AND FUNGAL MOLECULES THAT EXTEND LIFESPAN IN *C. ELEGANS*

A survey of the literature indicates that a large number of molecules and extracts from plants and fungi extend the lifespan of *C. elegans* (**Table 1**). Many of these natural substances are consumed in the human diet, and are found in vegetables, fruits, mushrooms, spices, tea, coffee and wine, while other extracts are derived from herbal and fungal remedies used in traditional Chinese medicine (e.g., *Ganoderma lucidum, Ginkgo biloba*, and *Rhodiola rosea*). Some pharmaceutical drugs were originally derived from plants and fungi, such as acetylsalicylic acid (aspirin), lovastatin and metformin, as well as molecules that were isolated from herbal remedies, including celastrol, huperzine A and triptolide (**Table 1**). In addition, many of the plant and fungal extracts and molecules included here are used as dietary supplements (e.g., *Antrodia cinnamomea*, glucosamine, propolis, quercetin and resveratrol).

**TABLE 1.** Examples of naturally-occurring substances and related pharmaceutical drugs that extend *C. elegans* lifespan. The “Mechanism” column displays modulation of specific cellular components (e.g., DAF-16↑, SOD-1↑, ROS↓) or involvement of particular genes, proteins and enzymes (e.g., DAF-2, OSR-1, Sir-2.1). In the “Lifespan” column, the parentheses indicate that lifespan assays were performed in the presence of cellular stress such as high glucose, heat or paraquat; in some studies, extension of “median” lifespan was reported. Only the highest increase in mean, median or maximum lifespan is shown. Abbreviations: AAK-2, 5' adenosine-monophosphate-activated protein kinase catalytic subunit alpha 2; AGE-1, phosphatidylinositol 3-kinase age 1; AGEs, advanced glycation endproducts; AMPK, 5'-adenosine-monophosphate-activated protein kinase; CBP-1, calcineurin-binding protein-1; DAF, abnormal dauer formation protein; EGCG, epigallocatechin gallate; FOX, forkhead box; GLP-1, abnormal germ line proliferation; HSF-1, heat shock factor 1; HSP, heat-shock protein; MDA, malondialdehyde; ND, not determined; NDGA, nordihydroguaiaretic acid; OSR-1, odd-skipped-related protein-1; ROS, reactive oxygen species; Sir, sirtuin; SKN-1, skinhead protein 1; SOD, superoxide dismutase; TOR, target of rapamycin; UPR^mit^, mitochondrial unfolded protein response.

**Substance**	**Chemical Class**	**Source**	**Mechanism (or Gene Involved)**	**Mean Lifespan**	**Maximum Lifespan**	**Ref.**
Acetylsalicylic acid (aspirin)	Organic acid	Analgesic drug (derived from willow bark)	AAK-2/AMPK↑, DAF-16↑, SOD-3↑, ROS↓	+23% (ROS)		[21, 22]
Antcin M	Terpenoid	*Antrodia cinnamomea*	ROS↓	+7%		[47]
Aspalathin	Chalcone glycoside	Rooibos tea	DAF-16↑, ROS↓	+24% (high glucose only)		[48]
Baicalein	Flavonoid	*Scutellaria baicalensis*	SKN-1↑	+45%	+24%	[49, 50]
Betalains	Indole	Opuntia fruit	ROS↓	+34%		[51]
Boeravinone B	Rotenoid	*Boerhaavia diffusa*	DAF-16↑, SKN-1↑	+28%		[52]
Brazilin	Flavonoid	*Caesalpinia sappan*	DAF-16↑, HSP-16.2↓, SOD-3↑, ROS↓	+18%		[53]
Caffeic acid	Polyphenol	Plants	DAF-16↑, Sir-2.1, OSR-1	+15%		[54]
Caffeic acid phenyl ester	Polyphenol	Propolis	DAF-16↑	+9% (median)	+17%	[55]
Caffeine	Alkaloid	Coffee	DAF-16↑, CBP-1	+37%	+52%	[19, 56–58]
Calycosin	Isoflavone	*Astragalus membranaceus*	DAF-2, DAF-16↑	+25%		[59]
Carnosic acid	Terpenoid	*Rosmarinus officinalis*	SOD-3↑, SKN-1↑, HSF-1↑	+16%	+22%	[60]
Carnosol	Terpenoid	*R. officinalis*	SOD-3↑, ROS↓	+19%	+26%	[35]
Catechin	Flavonoid	Green tea	DAF-2	+15%		[61, 62]
Celastrol	Terpenoid	*Tripterygium wilfordii*	ND	+17%		[63]
Chlorogenic acid	Polyphenol	Coffee	DAF-2, DAF-16↑, SKN-1↑	+20%		[34]
Chlorophyll	Chlorin	Vegetables	DAF-16↑	+26%		[64]
Curcumin	Polyphenol	Turmeric	Sir-2.1, OSR-1	+55% (median)		[65, 66]
Damaurone D	Flavonoid	Damask rose	DAF-2, DAF-16↑, SOD-3↑	+17%	+21%	[67]
Dehydroabietic acid	Terpenoid	Conifer resin	Sir-2.1	+16%		[68]
Diallyl trisulfide	Organosulfur	Garlic	SKN-1↑	+13%		[69]
Diosgenin	Terpenoid	Plants	DAF-16↑, SOD-3↑	+20%		[70]
4,4'-Dimethoxychalcone	Chalcone	*Angelica keiskei koidzumi*	Autophagy↑	+20% (median)		[71]
Emodin	Anthraquinone	Rhubarb, buckthorn	Sir-2.1, DAF-16↑	+20%		[77]
Ellagic acid	Phenol	Fruits	DAF-16↑	+11%		[62, 78]
Ferulsinaic acid	Organic acid	Ferula plants	AGEs↓, ROS↓	+18%	+42%	[79]
Fisetin	Flavonoid	Fruits, vegetables	DAF-16↑, ROS↓	+6% (heat)		[80]
Flavonoids	Flavonoid	Onion	ND	+20%		[17]
Fruit extract	Mixture	Apple	ND	+39%	+25%	[81]
Fruit extract	Mixture	Blueberry	DAF-16↑, SKN-1↑, SOD-3↑	+44%	+24%	[82]
Fruit extract	Mixture	Mulberry	DAF-16↑, Sir-2.1	+20%	+9%	[83]
Fruit extract	Mixture	Orange	DAF-16↑, SOD-3↑, ROS↓	+26%	+26%	[84]
Fruit extract	Mixture	Pomegranate	DAF-16↑	+56%	+36%	[78]
Fruit extract	Mixture	Purple pitanga	DAF-16↑	ND		[85]
Fungal extract	Mixture	*Ganoderma lucidum*	GLP-1	+36%	+12%	[86]
Gallic acid	Phenolic acid	Fruits	ND	+12%		[62]
Genistein	Isoflavone	Soybean, coffee	SOD-3↑, HSP-16.2↑	+28%		[87]
Glucosamine	Amino sugar	Dietary supplement (can be isolated from wheat or corn)	AAK-2/AMPK↑, mitochondrial biogenesis↑, autophagy↑	+30%		[30, 88]
Glaucarubinone	Degraded terpenoid	Simaroubaceae plants	Cellular respiration↑	+8%	+8%	[89]
Huperzine A	Alkaloid	*Huperzia serrata*	ND	+13%		[90]
10-Hydroxy-2-decenoic acid	Organic acid	Royal jelly	ND	+12%	+21%	[91]
Icariin	Flavonoid glycoside	*Epimedium brevicornum*	DAF-16↑	+21%		[92]
Icariside II	Flavonoid glycoside	*E. brevicornum*	DAF-16↑, HSP-12.3↓	+31%		[92]
Isorhamnetin	Flavonoid	Onion	ROS↓	+16%	+16%	[93]
Kaempferol	Flavonoid	Fruits, vegetables	DAF-16↑, ROS↓	+10% (heat)	+7%	[80, 94]
Laricitrin	Flavonoid	Red grapes and wine	DAF-16↑	+55%		[95]
Lignans	Polyphenol	*Arctium lappa*	DAF-16↑	+25%		[96]
Lovastatin	Lactone	Mushrooms	DAF-16↑	+25%		[97]
Metformin	Biguanide	*Anti-diabetic drug (derived from French lilac)*	AAK-2/AMPK↑, TOR↓, SKN-1↑, methionine↓, agmatine↑	+40% (median)		[37, 98–100]
Monascin	Azaphilonoid	*Monascus purpureus*	DAF-16↑, SOD-1↑, HSP-16.2↑	+29% (CL2006 strain)		[101]
Myricetin	Flavonoid	Fruits, vegetables	DAF-16↑, ROS↓, Sir-2.1	+48%	+22%	[94, 95, 102, 103]
Myricetin-trimethylether	Flavonoid	Bridelia plant	DAF-16↑	+54%		[95]
Naphthazarin	Naphthoquinone	Plants	SKN-1↑	+13%	+25%	[18]
NDGA	Polyphenol	*Larrea tridentata*	Autophagy↑	+21% (median)		[104]
5'-Octanoyl salicylic acid	Organic acid	Skin exfoliating drug (aspirin derivative)	AAK-2/AMPK↑, TOR↓, autophagy↑, UPR^mit^↑	+19%	+12%	[105]
Oleanolic acid	Terpenoid	Plants	DAF-16↑, ROS↓	+17%		[106]
Oxoline	Naphthoquinone	Plants	ND	+15%	+10%	[18]
Piceatannol	Stilbenoid	Red grape, wine	DAF-2, DAF-16↑, Sir-2.1	+18% (median)		[107]
Plant extract	Mixture	*Alpinia zerumbet*	SOD-3↑, HSP-16.2↑	+23%	+61%	[108]
Plant extract	Mixture	*Anacardium occidentale*	DAF-16↑, SKN-1↑, SOD-3↑	+20%		[109]
Plant extract	Mixture	*Betula utilis*	DAF-16↑, HSF-1↑, SKN-1↑, ROS↓	+36%		[110]
Plant extract	Mixture	Black tea	ND	ND		[111]
Plant extract	Mixture	*Caesalpinia mimosoides*	DAF-16↑, ROS↓	+4%		[112]
Plant extract	Mixture	*Damnacanthus officinarum*	ND	+10–30%		[113]
Plant extract	Mixture	*Dioscorea alata*	HSP-16.2↑, SKN-1↑	+28%		[114]
Plant extract	Mixture	*Eleutherococcus senticosus*	DAF-16↑	+16%	+12%	[25]
Plant extract	Mixture	*Garlic*	DAF-16↑	+21%		[115]
Plant extract	Mixture	*Ginkgo biloba*	ROS↓	+8% (median)		[116, 117]
Plant extract	Mixture	*Glochidion zeylanicum*	DAF-16↑, SKN-1↑, SOD-3↑, HSP-16.2↓	+10%		[118]
Plant extract	Mixture	Green tea	EAT-2	ND		[111]
Plant extract	Mixture	Guarana	DAF-16↑	+14%		[119]
Plant extract	Mixture	*Hibiscus sabdariffa*	DAF-16↑, SKN-1↑	+24%		[120]
Plant extract	Mixture	*Lonicera japonica*	DAF-2, DAF-16↑, SOD-3↑, ROS↓	+22%		[121]
Plant extract	Mixture	*Pu-er tea*	ND	ND		[111]
Plant extract	Mixture	*Ribes fasciculatum*	DAF-2, AGE-1, DAF-16↑, Sir-2.1, SOD↑, ROS↓	+16%	+19%	[122]
Plant extract	Mixture	*Rhodiola rosea*	DAF-16↑	+15%	+12%	[25]
Plant extract	Mixture	Rooibos tea	HSP-16.2↓	+23% (high glucose only)		[48]
Plant extract	Mixture	Turkish medicinal plants	ND	+24%		[123]
Plant extract	Mixture	*Viscum album coloratum*	Sir2	+10%		[26]
Plumbagin	Naphthoquinone	*Plumbago zeylanica*	DAF-16↑, SKN-1↑	+12%	+13%	[18]
Polydatin	Stilbenoid glycoside	Grape	DAF-16↑, SOD-3↑	+31%		[124]
Polysaccharides	Polysaccharide	*A. membranaceus*	DAF-16↑	+20% (median)		[125]
Polysaccharides	Polysaccharide	*Auricularia auricular*	DAF-16↑, SKN-1↑, Sir-2.1	-18%	+22%	[126]
Polysaccharides	Polysaccharide	*Chlorophytum borivilianum*	ND	+23%	(median)	[127]
Polysaccharides	Polysaccharide	*Cordyceps militaris*	ND	+17%		[128]
Polysaccharides	(lentinan)	Polysaccharide	*Lentinula edodes*	ND	+11%	[128]
Polysaccharides	Polysaccharide	*Panax notoginseng*	SOD↑, catalase↑, MDA↓	+21%		[129]
Polysaccharides	Polysaccharide	*G. lucidum*	DAF-16↑, autophagy↑	+44%	(median)	[130], unpublished data
Polysaccharides	Polysaccharide	*Rehmannia glutinosa*	DAF-16↑	ND		[131]
Polyphenols	Polyphenol	Apple	Sir-2.1	+12%		[132]
Polyphenols	Polyphenol	Blueberry	ROS↓, OSR-1, SEK-1↑	+28%	+14%	[133]
Polyphenols	Polyphenol	Cocoa	DAF-16↑, Sir-2.1	+17% (median)		[134]
Quercetin	Flavonoid	Vegetables	AGE-1, DAF-2, DAF-16↑, SEK-1↑	+15%	+18%	[54, 93,94, 135–138]
Quercetin-3-O-glucoside	Flavonoid glycoside	Vegetables	ND	+23%	+7%	[139]
Quinic acid	Polyol	*Uncaria tomentosa*	DAF-16↑, SOD-3↑	+7%		[140]
Reserpine	Alkaloid	Indian snakeroot, anti-hypertensive drug	Stress tolerance↑	+31%		[141]
Resveratrol	Stilbenoid	Red wine, dietary supplement	Sir-2.1, autophagy↑	+18%		[142–147]
Rosmarinic acid	Polyphenol	*R. officinalis*	DAF-16↑, OSR-1, SEK-1↑, Sir-2.1	+63%		[54,148]
Royal jelly	Mixture	*Dietary supplement*	DAF-16↑	+9%		[91]
S-allylcysteine	Organosulfur	Garlic	SKN-1↑	+17%		[149]
S-allylmercaptocysteine	Organosulfur	Garlic	SKN-1↑	+21%		[149]
Spermidine	Polyamine	Natto, mushrooms	Autophagy↑	+15%		[150]
Silymarin	Flavonolignan	Milk thistle	DAF-16↑, SOD-3↑, ROS↓	+18%		[151]
Simvastatin	Lactone	Cholesterol-lowering drug (derived from fungi)	ND	+13%		[97]
Syringetin	Flavonoid	Sichuan pepper	DAF-16↑	+36%		[95]
Tamarixetin	Flavonoid	*G. biloba*	ROS↓	+29% (median)		[93,116]
Tambulin	Flavonoid	*Zanthoxyllum aramatum*	DAF-16↑, SOD-1↑, SOD-3↑, ROS↓	+17%		[152]
Tannic acid	Polyphenol	Plants	SEK-1↑	+19%		[62,153]
Taurine	Amino sulfonic acid	Dietary supplement	ND	+11%		[154]
Theanine	Amino acid	Tea, dietary supplement	ND	+14%		[154,155]
Theophylline	Alkaloid	Coffee	ROS↓	+21%		[19]
Tocotrienols	Tocopherol	Fruits, vegetables	ROS↓	+20%		[156]
Tomatidine	Alkaloid	Unripe tomatoe	SKN-1↑	+7%		[157]
Trehalose	Disaccharide	Vegetables, mushrooms	DAF-2	+30%		[158]
Triptolide	Terpenoid	*T. wilfordii*	SOD-3↑, HSP-16.2↑, ROS↓	+20%	+16%	[159]
Ursolic acid	Terpenoid	Plants	SKN-1↑	+31%		[160]

**Figure 2:**
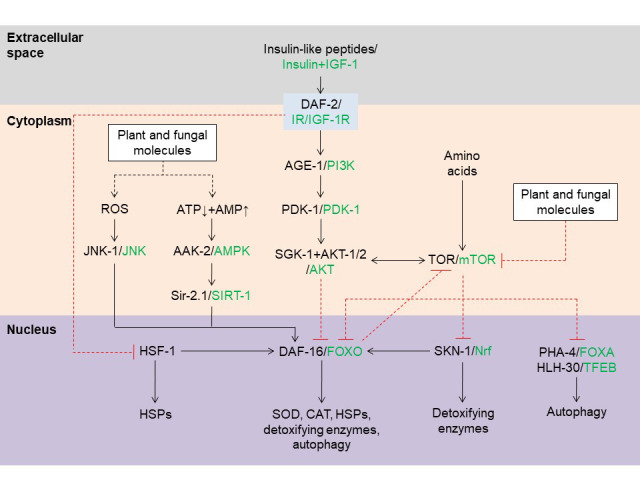
FIGURE 2: Aging-related pathways modulated by plant and fungal molecules in *C. elegans*. Plant and fungal molecules extend nematode lifespan by inducing the formation of ROS, by activating AAK-2/AMPK, or by inhibiting the insulin or TOR pathway. General cellular pathways are shown here, but variations may occur between cells of different tissues. Human protein homologs are given in green. Abbreviations: AGE-1, phosphatidylinositol 3-kinase age 1; AMP, adenosine monophosphate; ATP, adenosine triphosphate; AAK-2, 5' adenosine-monophosphate-activated protein kinase catalytic subunit alpha 2; AMPK, 5' adenosine-monophosphate-activated protein kinase; CAT, catalase; DAF, abnormal dauer formation protein; FOX, forkhead box; HLH-30, basic helix-loop-helix protein 30; HSF-1, heat-shock factor 1; HSPs, heat-shock proteins; IGF-1, insulin-like growth factor 1; IGF-1R, insulin-like growth factor 1 receptor; IR, insulin receptor; JNK, c-Jun N-terminal kinase; mTOR, mammalian target of rapamycin; Nrf, nuclear factor erythroid 2-related factor; PDK-1, 3' phosphoinositide-dependent protein kinase 1; PHA-4, defective pharyngeal development protein 4; PI3K, phosphoinositide 3-kinase; ROS, reactive oxygen species; SGK-1, serum and glucocorticoid-regulated kinase-1; Sir-2.1, sirtuin 2.1; SIRT-1, sirtuin 1; SKN-1, skinhead 1; SOD, superoxide dismutase; TFEB, HLH transcription factor EB; TOR, target of rapamycin.

While many natural substances can extend the lifespan of nematodes, they act by regulating a small set of cellular pathways (**Table 1** and **Figure 2**). One of the main cellular pathways that control *C. elegans* lifespan is the insulin pathway induced by food intake [12, 15]. This pathway consists of DAF-2 (the homolog of the human insulin receptor), several conserved protein kinases, and DAF-16 (the sole homolog of the FOXO family of transcription factors; **Figure 2**). In nematodes, insulin-like peptides bind to DAF-2 and induce intracellular signaling that leads to phosphorylation of DAF-16, thereby sequestering the transcription factor in the cytoplasm; in the absence of insulin-like peptides and DAF-2 signaling, as occurs when food is scarce, DAF-16 migrates into the nucleus where it induces expression of several genes including heat-shock proteins (HSPs) and antioxidant enzymes like superoxide dismutase (SOD) and catalase (CAT), as well as autophagy-related proteins (**Figure 2**) [12, 15].

Another pathway activated by food intake involves the target of rapamycin (TOR), which is activated by nutrients and amino acids (**Figure 2**). Inhibition of TOR activates skinhead 1 (SKN-1), the homolog of nuclear factor erythroid-2-related factor (Nrf) proteins, and defective pharyngeal development protein 4 (PHA-4), the homolog of human FOXA proteins, leading to expression of detoxifying enzymes and activation of autophagy, respectively [12]. TOR inhibition also activates autophagy by inducing basic helix-loop-helix protein 30 (HLH-30), the homolog of HLH transcription factor EB (TFEB) [16]. In addition, the nicotinamide adenine dinucleotide (NAD^+^)-dependent protein deacetylase Sir-2.1, the homolog of human sirtuin-1, induces anti-aging effects at least in part by stimulating DAF-16 activity (**Figure 2**).

Phytochemicals were previously believed to produce beneficial effects on health and longevity mainly by acting as antioxidants that scavenge reactive oxygen species (ROS). However, several lines of evidence indicate that these molecules may act in other ways, notably by inducing stress resistance and anti-aging pathways [5, 6]. Accordingly, the antioxidant properties of phytochemicals *in vitro* do not correlate with anti-aging effects in *C. elegans* [17]. Moreover, some phytochemicals can, instead, extend *C. elegans* lifespan by inducing ROS formation, which in turn leads to expression of SKN-1 and antioxidant enzymes that protect from oxidative stress by inactivating ROS [18]. For example, theophylline, a methylxanthine compound found in cocoa, chocolate, tea and guarana, slightly increases ROS levels in *C. elegans*, which prolongs lifespan and increases resistance to the ROS-producer juglone [19]. Plant molecules that induce ROS formation may activate c-Jun N-terminal kinase 1 (JNK-1) and DAF-16 (**Figure 2**). Other phytochemicals activate SKN-1 and lead to reduction of ROS in a similar manner (**Table 1**).

While several plant-derived compounds extend lifespan in nematodes, conflicting results have been obtained in some cases, possibly due to differences in study design or experimental conditions. For instance, the Caenorhabditis Intervention Testing Program, which aims to identify anti-aging compounds that prolong lifespan in genetically diverse cohorts of *C. elegans*, reported that aspirin does not extend lifespan [20], contradicting the results of previous studies [21, 22].

## LIFESPAN EXTENSION OCCURS VIA HORMESIS

It has been proposed that many molecules derived from plants and fungi induce stress resistance and defense mechanisms via hormesis, i.e., which posits that cellular stress that is detrimental at high intensity can produce health benefits at low intensity [5, 6, 23]. By activating autophagy, mitochondrial biogenesis and expression of antioxidant and detoxifying enzymes, plant and fungal products reduce cellular damage and improve cellular functions, thus reducing aging and extending longevity [6]. This mechanism is consistent with the concept that, under conditions of stress such as CR, the organism allocates more energy for resistance and survival, instead of growth and reproduction [24].

The hormetic dose-dependence is observed in several studies listed in **Table 1**. For example, treatment of *C. elegans* with an extract of Siberian ginseng (*Eleutherococcus senticosus*) extends mean lifespan by 5% at low dose (100 μg/ml) and by 16% at intermediate dose (250 μg/ml), whereas the same extract reduces mean lifespan by 23% at high dose (2,500 μg/ml) [25]. Similar hormetic dose-responses involving lifespan extension at low doses and lifespan shortening at high doses were obtained for plant extracts of *Rhodiola rosea* [25] and mistletoe [26], and for the tea polyphenol epigallocatechin gallate (EGCG) [27], to name a few. However, this dose dependence has been largely overlooked in many studies, while in other cases, a relatively narrow range of concentrations tested may have prevented the observation of hormetic dose-responses.

Another observation suggesting that plant and fungal compounds extend lifespan via hormesis is the fact that stress resistance pathways are activated in the treated worms. Thus, many plant and fungal compounds that include 4,4'-dimethoxychalcone, glucosamine, nordihydroguaiaretic acid (NDGA), resveratrol and spermidine extend the lifespan of *C. elegans* by activating autophagy (**Table 1** and **Figure 2**), which in itself is a typical cellular response to stress [6, 28]. We also observed that polysaccharides isolated from the medicinal fungus *G. lucidum* extend the lifespan of *C. elegans* by inducing autophagy (unpublished data). In addition, several plant and fungal products increase the levels of HSPs and antioxidant and detoxifying enzymes (**Table 1**), reflecting a cellular response that aims to maintain homeostasis in response to stress.

Plant and fungal compounds can also induce mitochondrial biogenesis via a process referred to as “mitohormesis” [29]. High levels of ROS usually induce cellular damage, but as mentioned above some phytochemicals can induce the formation of low levels of ROS which in turn induce stress resistance mechanisms. In this case, cells respond by forming new mitochondria which in turn may improve cellular function and longevity. Examples of natural compounds that act this way in nematodes include EGCG [27] and glucosamine [30] (**Table 1**). Of note, excess intake of antioxidants such as vitamins C and E may reduce the health benefits of anti-aging interventions like exercise in humans by preventing mitohormesis [29].

In the studies consulted, plant and fungal extracts and molecules extend mean or median lifespan of nematodes by an average of 4 to 63% (**Table 1**). These lifespan extensions are consistent with the hormetic effects observed in a large number of studies reporting the responses of microbes, plants and animals to various forms of biological stress, in which maximum effects of 20–90% above control were reported [31]. While hormetic responses may be relatively modest in magnitude, they are nevertheless highly significant in view of their overall impact on health and longevity.

Of note, only some plant or fungal substances increase maximum lifespan, producing increases ranging from 7 to 68% (**Table 1**). While a description of the effects on maximum lifespan may have been omitted in some studies, this observation nonetheless suggests that the treatments may reduce the number of deaths in adult worms at some point in time but fail to extend the lifespan of old worms. Given that hormetic effects have been attributed to an overcompensation of homeostasis-regulating mechanisms and may thus rely on the capacity to maintain homeostasis [32], the absence of effects on maximum lifespan in some studies may indicate that very old individuals are unable to maintain homeostasis in response to biological stress, possibly due to a loss of resilience. Consistent with this possibility, feeding *C. elegans* with metformin late in life produces toxic effects and reduces lifespan by exacerbating age-related mitochondrial dysfunction [33], unlike the lifespan-enhancing effects of metformin seen in younger worms. Similarly, the lifespan-extension effects of EGCG decline with age [27]. This indicates that CR mimetics—and possibly other anti-aging interventions that work through hormesis—may be ineffective and even detrimental in very old individuals.

## EFFECTS OF NATURAL PRODUCTS ON HEALTHSPAN VIA THE GUT MICROBIOTA

While studies in *C. elegans* have focused on extension of lifespan, many reports showed that natural substances that extend lifespan also produce beneficial effects on healthspan. For instance, plant-derived polyphenols such as chlorogenic acid, which is found in vegetables and coffee, improve insulin sensitivity and mobility in the treated worms [34]. Similarly, carnosic acid, a diterpene compound isolated from rosemary (*Rosmarinus officinalis*), improves mobility and aging-related pigmentation and neurodegeneration in nematodes [35]. These observations are consistent with the view that interventions that prolong lifespan may also improve physiological functions and reduce development of chronic disease.

Recent studies suggest that some of the beneficial effects on health and longevity in nematodes may take place via modulation of the gut microbiota. A key study showed that *Escherichia coli* mutants deficient in some biochemical components can extend nematode lifespan [36]. This study reported that production of the polysaccharide colanic acid by gut bacteria can extend lifespan and reduce age-related pathologies by inducing the unfolded protein response in the host. Similarly, metformin can extend lifespan and regulate host lipid metabolism via production of the metabolite agmatine by the gut microbiota [37]. Other studies showed that a strain of the probiotic *Lactobacillus rhamnosus* [38] or *Weissella* bacteria activated the DAF-16 pathway and extended *C. elegans* lifespan compared to feeding with *E. coli* [39]. However, these results may also be partially explained by the observation that *E. coli* becomes pathogenic for old worms and feeding with less pathogenic bacteria may therefore extend nematode lifespan [40]. Given that major differences exist between gut microbiota composition in *C. elegans* and humans—including the fact that the gut microbiota in nematodes studied *in vitro* usually consists of a single bacterial species provided as food—further studies are needed to assess the relevance of these observations in humans.

## CHALLENGES AND OPPORTUNITIES

Our overview indicates that many plant and fruit extracts derived from blueberries to garlic, as well as plant molecules such as chlorophyll and caffeine, extend the lifespan of *C. elegans* (**Table 1**). Yet, many factors may partially limit the relevance of these findings for humans, including major differences in physiology and metabolism. Health and longevity in humans depend on complex interactions between genetic background, lifestyle and diet, which can hardly be reproduced in experimental settings. It is likely that common lifestyle habits such as overeating, smoking, sedentarity, alcohol intake, stress and poor sleep, as well as environmental factors such as pollution, ultraviolet light and toxins, may reduce, suppress or even reverse the beneficial effects of phytochemicals and CR mimetics on health and longevity. Moreover, the appropriate concentrations and treatment schedule required to produce optimal health benefits remains largely unknown. The observations reported here also suggest that CR mimetics may become ineffective and even detrimental at very old age, therefore requiring the identification of optimal doses for older individuals and the development of new ways to monitor homeostasis and resilience.

Nonetheless, several epidemiological studies suggest that some of the plant-derived molecules described here may reduce human mortality and chronic diseases in humans. For instance, individuals who regularly consume coffee—arguably the highest source of polyphenols and caffeine in the human diet—live longer and show a reduced incidence of cancer, cardiovascular disease and Alzheimer's disease compared with non-consumers [41, 42]. Similarly, people who regularly take metformin [43] or glucosamine [44, 45], as well as those who have a higher dietary intake of spermidine [46], live longer than non-users or controls. Finally, many CR mimetics derived from natural sources and studied in *C. elegans*, including quercetin, resveratrol and spermidine, have shown promising results in clinical trials [8]. It thus becomes a matter of when and how—as opposed to if—these plant and fungal molecules can be used in humans.

## Abbreviations:

CR: – caloric restriction;
EGCG: – epigallocatechin gallate;
HSP: – heat shock protein;
ROS: – reactive oxygen species;
TOR: – target of rapamycin.
